# Supplementary material for: Efficient Learning Strategy of Chinese Characters Based on Network Approach
Source: PLoS One. 2013 Aug 21;8(8):e69745. doi: 10.1371/journal.pone.0069745 (PMC3749196; doi:10.1371/journal.pone.0069745)
Supplement: File S1 — Supplementary information for efficient learning strategy of Chinese characters based on network approach. (PDF) [file pone.0069745.s001.pdf]

Supplementary information for efficient learning strategy of Chinese characters based on network approach

Xiaoyong Yan[1,2], Ying Fan[1], Zengru Di[1], Shlomo Havlin[4], Jinshan Wu[1,*]

1 School of Systems Science, Beijing Normal University, Beijing, 100875, P.R. China

2 Center for Complex Systems Research, Shijiazhuang Tiedao University, Shijiazhuang 050043, P.R. China

3 Department of Physics, Bar-Ilan University, Ramat-Gan 52900, Israel

∗ E-mail: Corresponding jinshanw@bnu.edu.cn

1 Data and methods

1.1 Decomposition of Chinese characters

According to "Liu Shu" (six ways of creating Chinese characters [25]), ideally when sub-characters are combined to form a character the compound character should be connected to its sub-characters either via their meanings or pronunciations. Thus, Chinese characters are usually meaningfully and coherently connected to each other. Let us start from the bottom of Figure 1 in the main text. The four characters are '一'(one), '大' (person, big), '心' (heart), '水' (water). These characters closely resemble the shapes or characteristics of the objects to which they refer, though their forms today might not hold as much of a resemblance as their ancient forms. One can compare the modern simplified Chinese character against their ancient Zhuanti forms in the figures. Such characters are called pictographic (Xiangxing) characters.

Initially, the character '天' (sky) refers to the head, the primary part of a person, by placing a bar over the character '大' (person, big). The meaning later developed and became the sky, heaven and god, i.e. the primary part of everything as ancient Chinese people believed. This way of forming new characters from radical parts is called "simple" ideogram (Zhishi) or "combination character" ideogram (Huiyi). These two mechanisms are in fact slightly different in that the first is based on only one radical part, usually with only a very simple additional stroke while the second usually involves two radical parts. For a character formed by these two principles, its meaning usually can be read out intuitively from the combination. For example, the character '森' (forest) mentioned in the introduction of the main text follows the principle of "combined" ideogram: it is a stack of three '木' (tree). However, in this work, we will not distinguish the two mechanisms.

The character '忝' (ashamed) is a compound character of '天' and '心'. It follows a different principle, which later became popular in forming new Chinese characters, the so-called pictophonetic formation (Xingsheng). Here, '忝' and '天' have exactly the same pronunciation, and the meaning of '忝' refers to a psychological phenomenon, which was believed to be related to '心' (heart). The same pictophonetic relation holds among 添' (add), '忝'and '水' (water): the first two share the pronunciation while the last part '水' is remotely connected to the meaning of '添'. In Figure 1 of the main text, we also notice that the characters '水' and '心' also form the characters '沁' (seep). The character '沁' follows also the pictophonetic formation. It is quite common that some basic characters are used in quite a few composed characters.

Here we have demonstrated four of the six principles. The other two are phonetic loan (Jiajie) and derivative cognates (Zhuanzhu). Those two principles are more on usage of characters but not on creating new characters. It is not our focus of this work to discuss various ways of usages of Chinese characters. Following the above general principles, our decompositions of characters are based primarily on Ref.[15,16,25]. The first is a standard reference, where the six principles were first explicitly discussed, in Chinese etymology studies, and the last two are regarded as developments of the first, mainly due to discoveries of new materials, including Oracle characters (Jiaguwen) and Bronze characters(Jinwen).

1.2 Construction of character network

Starting from 3500 characters, our network ends up with a giant component of 3687 nodes and 7024 links, plus 15 isolated nodes. Why do we have more nodes than the total number of characters we start with? In our decomposition, we find some sub-characters beyond the set of the most used 3500 characters. Sometimes, such sub-characters are just variations of their normal forms. In this case, we merge the characters. The situation becomes more complicated when a radical whose corresponding normal form is not within the most-used set. In such cases, we add the "never-independent characters" as extra nodes in the network. For example, '秀' is such a rarely used character, but we keep it in our network. Other examples includes radicals which are popular and play important roles in forming many compound characters but cannot be used as independent characters. We also include these in our network. In some special cases, some characters have same sub-characters (*e.g.* '林' has two sub-characters '木'), we combine the sub-characters to one node and link the sub-character to the character with an unweighted edge.

See Figure 2 in the main text for the full map of structural relations among Chinese characters.

1.3 Additional explanation of definition of learning cost

We define the learning cost of a character for a student to be the sum of the number of sub-characters and the learning cost (calculated recursively) of the unlearned sub-characters at his current stage. The recursive definition seems to imply that when a student is learning a compound character, he has to recognize first the sub-characters. However, the dynamic process is only a fictitious process used to represent the difficulty that the student faces in learning the character. It does not means the learning process is indeed as such. Recall from the main text total cost of learning '透' before '秀' is $4 = 2 + 2$, which is from the fact that it has 2 sub-characters and also from the fact that cost of learning the unknown '秀' is 2. Therefore, determining cost of learning '透' first obviously involves cost of learning '秀'. However, this does not imply that the student should have known '秀' after acquiring '透'. If it happens so that the next time the student must learn '秀', then the learning cost of '秀' is still 2 even he had learned '透' before. Thus the total learning cost of the two characters following the order of '透' $\rightarrow$ '秀' is 6.

Of course, if the student learned the character '透' meaningfully, i.e. when he learns the character '透', he indeed learns also the relation between '透' and '秀' (also the meaning of '秀') explicitly from his books or his instructors, then the total cost for him to learn both characters is in fact 4 (no cost for learning '秀'), which is the same cost of learning both characters in the order of '秀' $\rightarrow$ '透'. Therefore, learning closely connected characters together at the same time and learning them meaningfully would reduce the cost. Therefore, one might conclude that our definition of learning cost does not apply to such meaningful learning. However, for this we would argue that such meaningful learning has implicitly used the optimal learning orders, learning the two characters simultaneously and meaningfully is equivalent to learning them according to the proper order.

Another problem related to our definition of learning cost is that we treated the number of sub-characters and the cost of unlearned sub-characters equally. This can be questioned and should be investigated further. For example, one might introduce a parameter to rescale the number of sub-characters and then sum the two together. For simplicity, we have not yet discussed this issue. Finding the proper value of such parameters from empirical studies and then comparing performance of those learning orders again using the new definition of cost should be an interesting topic.

2 Supplemental Results

At last, we provide the two important lists of characters as final results of our network-based analysis of Chinese characters. First is the adjacency list of the network of characters. The first character of

every line is the starting point of links and all other characters in the same line are the ending point of the links, meaning the first character is a part of everyone of the other characters. Second is the order of Chinese characters listed according to the calculated DNW centrality. This list includes all 3500 characters and $b = 0.5$ is used in the calculation of DNW. In the main text, when 1775 characters are used as the learning target, we find the optimal value of parameter b is $b = 0.35$. Repeating the same analysis for all 3500 characters, we find that learning efficiency is higher when $b = 0.5$ is used instead of $b = 0.35$. Here the list is produced when we consider the whole set of most used characters as the learning goal. Those two lists are included in the SI of this publication. They are also provided on our own still developing website on Chinese learning at http://www.learnm.org/data/adjlist.txt and http://www.learnm.org/data/DNWorder.txt.
